# Triangulating the New Frontier of Health Geo-Data: Assessing Tick-Borne Disease Risk as an Occupational Hazard among Vulnerable Populations

**DOI:** 10.3390/ijerph19159449

**Published:** 2022-08-02

**Authors:** Sarah P. Maxwell, Connie L. McNeely, Chris Brooks, Kevin Thomas

**Affiliations:** 1 School of Economic, Political & Policy Sciences, University of Texas at Dallas, Richardson, TX 75080, USA; 2 Center for Science, Technology, and Innovation Policy, George Mason University, Fairfax, VA 22030, USA; cmcneely@gmu.edu; 3 Laboratory for Human Neurobiology, Boston University School of Medicine, Boston, MA 02118, USA; crbrooks@bu.edu (C.B.); kipthoma@bu.edu (K.T.)

**Keywords:** vulnerable populations, tick-borne disease surveillance, geo-data, occupational hazards, triangulation, migrant and seasonal workers

## Abstract

Determining interventions to combat disease often requires complex analyses of spatial-temporal data to improve health outcomes. For some vulnerable populations, obtaining sufficient data for related analyses is especially difficult, thus exacerbating related healthcare, research, and public health efforts. In the United States (U.S.), migrant and seasonal workers are especially affected in this regard, with data on health interventions and outcomes largely absent from official sources. In response, this study offers a multi-modal approach that involves triangulating geographically specified health data that incorporate reports on canine tick species, Lyme disease (LD) incidence, and patient symptom severity indicating potential subsequent disease burden. Spatial alignment of data at the U.S. county level was used to reveal and better understand tick-borne disease (TBD) incidence and risk among the identified populations. Survey data from migrant and seasonal workers in Texas were employed to determine TBD risk based on symptoms, occupations, and locations. Respondents who were found to have a higher likelihood of a TBD were also considerably more likely to report the most common symptoms of LD and other TBDs on the Horowitz Multiple Systemic Infectious Disease Syndrome Questionnaire. Those in the highly likely scoring group also reported more poor health and mental health days. Overall, a notable number of respondents (22%) were likely or highly likely to have a TBD, with particular relevance attributed to county of residence and living conditions. Also of note, almost a third of those reporting severe symptoms had received a previous Lyme disease diagnosis. These findings underscore the need for further surveillance among vulnerable populations at risk for TBDs.

## 1. Introduction

The United States (U.S.) is witnessing an increasing prevalence of vector-borne diseases, i.e., diseases caused by bacteria, parasites, and viruses transmitted to humans by vectors. Tick vectors in particular are spreading diseases across a rapidly expanding geographic scale and are reaching areas of the country previously considered non-endemic. Additionally, with antimicrobial resistance on the rise in the U.S., coupled with unreliable testing of variable quality, some patients suffering from tick-borne diseases (TBDs) likely lack adequate detection and treatment options. This is even more problematic since many TBDs, such as Lyme disease (LD), remain largely on the fringes of clearly defined treatment protocols and adequate surveillance [1]. Between 2000 and 2018, there were an estimated 500,000 cases of LD alone in the United States [2]. The seriousness of this situation has prompted research and policy interest in more comprehensive approaches to assessing human TBD risk and related factors.

The distribution of vector-borne diseases is determined by a complex set of factors such that disease outcomes and effects can vary substantially across different groups, with some populations considered especially vulnerable due to, for example, occupational settings, poverty, and substandard living conditions that are associated with exposure to disease vectors. It is in this regard that seasonal and migrant workers in the U.S. are particularly at risk and are of critical interest given their outdoor occupations. Migrant and seasonal farmworkers are vital to the U.S. economy, yet they are among the most marginalized and underserved populations in the country, with a range of unmet socioeconomic and healthcare needs [3]. Indeed, around the world, such workers are engaged in jobs that are hazardous to their health [4]. They work for longer hours, and in worse conditions, and are subject to health disparities linked to environmental and occupational exposures and to various social determinants, e.g., poverty, language/cultural barriers, lack of access to quality health care, and documentation status [4]. Due to their occupational environments and working and living conditions, seasonal and migrant workers may be at increased risk for contracting a variety of viral, bacterial, fungal, and parasitic infections [3]. Along these lines, migrant and seasonal farmworkers in the U.S. may face disproportionally elevated risks for contracting TBDs. From a public health perspective, a large part of the problem also is that tick-bite incidence and risk rates among humans are generally poorly documented. The lack of available information on TBDs beyond gross aggregate state-level data has garnered attention from public health researchers, who have noted the need for more detailed data and improved communication between medical practitioners and their patients. In the U.S. (with the exception of LD, which is reported at the county level), official data from the Centers for Disease Control and Prevention (CDC) on TBDs are available principally at the state level, with endemic designations determined by relative aggregate indications. Yet, in many cases, vulnerable populations are potentially likely to be exposed to tick pathogens causing ehrlichiosis, for example, across geological scales for which there is insufficient detail for robust analysis with aggregate state-level data. This has been particularly noted in states such as Texas that is perceived as non-endemic, yet possess varied eco-systems within which county-level research has revealed higher indications of TBD risk [5]. As a result, a growing number of analysts are now attempting to assess human disease risk in varying geographic regions via multi-modal approaches employing various risk proxies, including entomological, canine, and patient self-reports of tick bites and concomitant diseases [5,6,7].

This study offers a systematic assessment and analysis of occupational information, symptom severity, and geographic location patterns to estimate TBD risk in rural communities comprised primarily of seasonal and migrant workers. An important aim is to identify effective alternatives for determining risk and related factors within the broader occupational populations of interest. Recognizing how various occupational and social factors affect health outcomes can contribute to epidemiological understanding of the development of diseases and provide valuable insights to mitigate their spread within vulnerable populations. “The ability to observe and document how the constraints and possibilities many people face on a daily basis can provide important data about the causes of disease. Without these data, researchers will not be able to identify the etiology of disease for the most disadvantaged of society and solutions will not emerge to solve these issues” [8].

Spatially sensitive analyses that include triangulation with other disease risk indicators, such as canine serological data and symptom severity reported by seasonal and migrant workers themselves, may provide a promising approach to TBD surveillance [5,7]. While some analysts and medical practitioners eschew self-reported data due to its inherent drawbacks (e.g., reporting bias, no biological samples), previous research on TBDs has suggested the utility of self-reported data, such as tick-bite encounters [5,6], through trend matching and association with official Centers for Disease Control and Prevention (CDC) reports [5,7]. This study applied related knowledge by integrating county-level indicators of disease risk with self-reported data of disease sequelae to achieve greater insights into TBD-related conditions and outcomes. This multimodal and integrative research is of importance for public health in light of the attention to geographical considerations in relation to regions based on environmental and ecological characteristics. Moreover, recent research has suggested the use of triangulation, referring to the use of multiple measures to define a construct [9], which combines factors from various sources and different levels of analysis, to develop and/or identify robust proxies of human TBD risk [5,6].

A primary aim of this research is to determine patterns of TBD incidence to provide information that can help guide policies and actions to reduce disease risk and promote health and wellbeing. Differences in geographical exposure to ticks in work environments underscore the need for research on the spread of TBDs among vulnerable populations who may be exposed to outdoor living and working conditions. Additionally, mapping available geo-data provides directions for identifying key implications for TBD risk and needs for further research on occupation-based targeting for disease control [10].

### 1.1. Human TBD Risk Issues

The expanding geographic range of tick abundance and activity based on, for example, changes in precipitation, vegetation type and distribution, seasons, and human behavior is associated with an increased risk of tick bites [11]. LD by itself is a significant health threat and one of the most frequently diagnosed tick-borne diseases in the world [12]. Caused by the bacterium *B. burgdorferi* in the U.S., LD is spread by *Ixodes* ticks that then pass it on to humans. Regarding their most basic impact, TBDs such as LD act to limit the physical and mental capabilities [13] and activities of workers, thus potentially hindering their work performance and quality of life. 

Human presence in habitats favorable to ticks and their hosts, outdoor activities, and climatic factors that support a wider distribution of tick vectors have enhanced the risk and impact of TBDs on humans (and animals, which subsequently increases exposure and the risk to humans) [12]. Along with research findings showing that differences in socioeconomic and environmental conditions are critical determinants of varying health outcomes [8], related disparities in health reflect “differences in incidence, prevalence, morbidity, mortality, and burden of diseases and other adverse health conditions that exist among specific population groups in the United States” [14]. Accordingly, factors such as “working in locations suitable for tick habitats” (e.g., with sufficient precipitation) are prevalent amongst migrant and seasonal workers and suggest a higher potential risk of TBDs and sequelae. In fact, recent studies revealed significantly higher risk and prevalence of LD amongst outdoor agriculture workers, who are considered one of the occupational groups most frequently affected by LD [11]. However, the actual numbers and prevalence of TBD incidence among these groups are unknown.

### 1.2. Analytical Perspective

The need to categorize and incorporate into analysis various kinds of data reflecting different types and levels of analysis demands a systematic and encompassing approach. Accordingly, a “grounded theory” approach is adopted here, involving the “use of multiple data sources converging on the same problem” where no one kind of data or technique of collection is sufficient on its own; it is a process of discovery based on observations from which theory emerges in explanation of the topic and population of concern [15,16]. More specifically, grounded theory involves the discovery of patterns in data [15] and, in this case, integrating finer-grained geo-data such as county-level indicators has implications for understanding tick exposure and disease outcomes among differing occupational groups of workers in agricultural communities.

Grounded theory entails iterative processes of comparison for determining behavioral and social patterns. As such, it is especially applicable here since emphasis is placed on determining relevant patterns and tendencies from various differentiated data. Based on this approach, this research includes multimodal data triangulation to engage and explore the various analytical factors that indicate TBD outcomes and risk. It builds on spatial data triangulation research to explore human TBD risk and concerns the grounding of theory development through data triangulation. Data triangulation refers to the use of different sources of data derived from, for example, analyzing people via surveys, group interactions, and as parts of collectivities. In this sense, data triangulation employs a systematic integration of data relative to selected persons, populations, and spatial–temporal settings [16,17].

## 2. Materials and Methods

This mixed-methods study uses geo-spatial data and survey responses from migrant farmworkers and other vulnerable participants to analyze human disease risk via numerous associated variables, including symptoms, self-perceived symptom severity, occupational risk factors, and geo-spatial indicator. Self-reports of vulnerable workers’ health and exposure to ticks are triangulated with other sources of official public health data, including CDC LD; Texas Department of State Health Service (DSHS) TBD reports; and canine serological reports of LD, ehrlichiosis, and anaplasmosis obtained via the Companion Animal Parasite Council (CAPC). One health approaches are key to triangulation since, no matter the geographic region, “where ticks are found, tick-borne diseases can present a threat to human and animal health” [18]. Triangulation of data points from official public health sources with survey respondent self-reports of disease and symptoms is a promising approach to assessing human TBD risk, and use of multi-layer thematic mapping provides a visual representation of corresponding human disease risk. For vulnerable populations, these mixed-method approaches are particularly important, as official data sources at the county level are lacking, resulting in vague and tenuous risk assessments by public health experts and medical providers for these populations.

In 2021, Spanish-speaking, IRB-trained nurses and health workers traveled to thirteen different sites in Texas to survey Spanish-speaking community members who were working in agricultural sites or who were attending community fairs in the area. These included co-op market sites to community events sponsored by the Consulado General de México en Dallas. The convenience sample included respondents who self-selected by volunteering to take the survey, and survey respondents were given a USD10 gift certificate upon completion of the survey. There was no reason to suspect respondents would be ill in any of the sites, i.e., the respondents were not individuals who reported or were known to have any symptoms, rashes, or tick-borne diseases. Nurses and health workers were trained to answer questions respondents might have, for example, describing an EM rash rather than simply asking if the respondent had ever had an EM rash. Surveys were administered in Spanish. Although agricultural workers were the intended target population, surveys were administered to individuals from a variety of occupations, including gardening, construction, and food service.

The targeted sites included counties across a spectrum of TBD risk based on official CDC LD data. Thirteen interview sites were targeted across nine counties from various Texan ecosystems. The nine counties contained a spectrum of TBD risk, based on CDC LD case data from 2000 to 2019 (total number of reported cases):

- Low risk (≤4 cases): Dallam County (0), Caldwell County (3), Jim Wells County (3), Star County (3), and Gregg County (4)
- Higher risk (>4 cases): Hays County (24), Hidalgo County (34), Bexar County (38), and Travis County (169)

The survey instrument included the full Horowitz Multiple Systemic Infectious Disease Syndrome (MSIDS) Questionnaire, or HMQ, in addition to questions to assess other aspects of the respondents’ lives, such as type of agriculture in which he or she worked. The HMQ scores symptoms associated with Lyme and other tick-borne diseases. Section 1 of the HMQ scores frequency of general symptoms such as headache, neck stiffness, poor short-term memory, confusion, and unexplained fevers, among many other symptoms reported in the medical literature. Section 2 of the survey focuses on issues related to tick-borne disease diagnoses, such as a previous tick bite, followed by flu-like symptoms and a prior diagnosis of chronic fatigue syndrome, for example. Section 3 asks about the number of poor physical and mental health days the respondent is experiencing. The HMQ was empirically validated through three studies, supporting its use as an effective and low cost tool in distinguishing healthy individuals from those with LD [13]. The survey expanded on Lyme and TBD symptoms to include questions related to occupation, migration patterns, and past diagnosis of Lyme disease. The survey was approved by the Ethics Committee under the Declaration of Helsinki Institutional Review Board Guidelines.

The HMQ was administered via the Qualtrics survey platform, which included an automatic scoring function to estimate TBD risk by applying published cutoff scores using the overall HMQ score: 0–20 = Not Likely; 21–36 = Possible; 36–62 = Likely; and ≥63 = Highly Likely [13]. The scoring guidelines were used to organize the results below. The four categories were condensed to three in the results below by combining the Likely and Highly Likely respondents (i.e., those with an HMQ score ≥ 36) into a single group. For triangulation purposes, maps with county-level data included any respondent who could possibly have LD or TBD (i.e., HMQ score ≥ 21). For all analyses, the Likely, Highly Likely, and Possible respondents were compared to those who were asymptomatic and not likely to have a TBD.

## 3. Results

**Figure 1:**
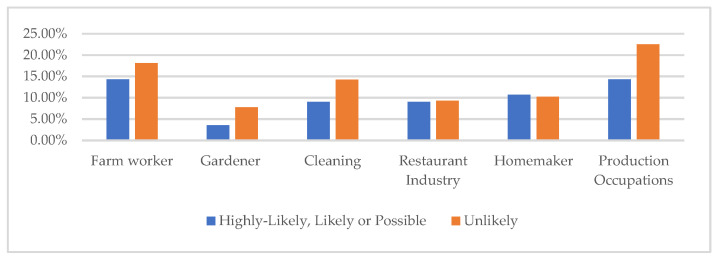
Occupation by HMQ score.

**Figure 2:**
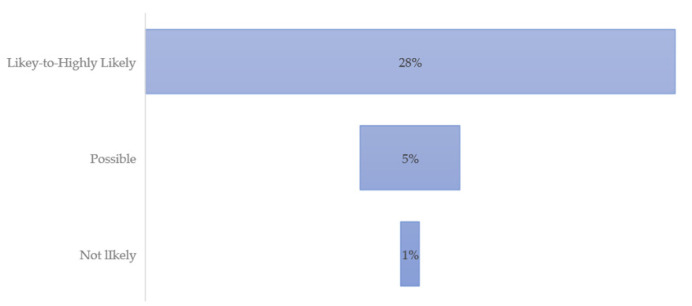
Percent of respondents reporting top five symptoms indicative of a TBD “All the Time”.

**Figure 3:**
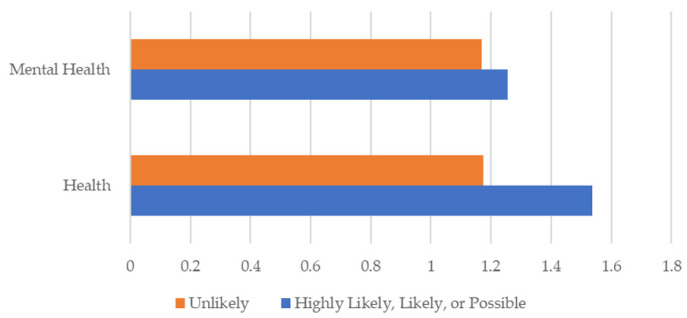
Average poor mental health and health within the last 30 days by scoring category.

**Figure 4:**
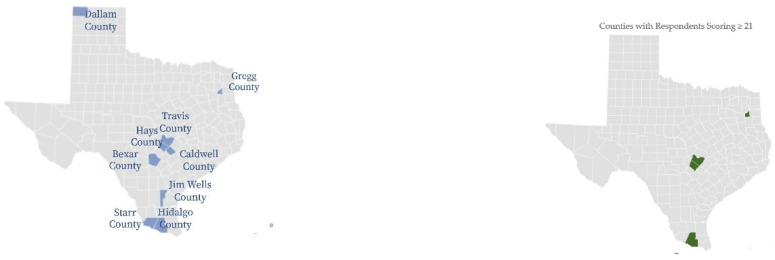
All survey respondent by counties and by counties with scores ≥ 21.

**Figure 5:**
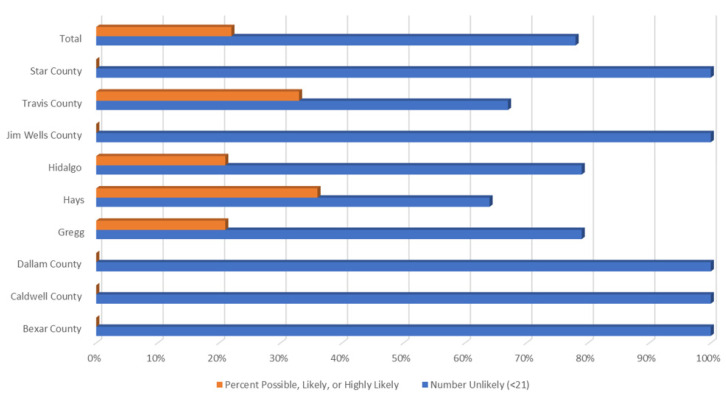
Percentage possible, likely, or highly likely, or not likely to have a TBD by county.

Using triangulation methods to establish patterns of illness and disease, the results include the survey respondents demographics (Table 1); occupations of respondents by symptom severity (Figure 1); percent of respondents reporting the top five symptoms indicative of a TBD “All the Time” (Figure 2); average poor mental health and health within the last 30 days by scoring category (Figure 3); survey respondent by counties with scores ≥ 21 (Figure 4); and percentages of those possible, likely, or highly likely to have a TBD vs. those who are unlikely, by county (Figure 5).

These indicators include official data on TBDs from the Texas Department of State Health Services (DSHS) and canine serological reports from the Companion Animal Parasite Council (CAPC). DSHS provided county-level data on LD, ehrlichiosis, chaffeesis and spotted fever rickettsiosis case rates in humans. In contrast to the state-level data from the CDC, DSHS’ county-level information affords an opportunity for a finer-grained public health analysis. Additionally, DSHS data contained additional information, such as whether the TBD was acquired locally versus out-of-state, better informing public health decision making. In some cases, TBD maps were used directly from the DSHS and respondent county data were overlaid on them. Cases not locally acquired were removed from the triangulation analysis and were also excluded from DSHS-prepared maps. The patterns were specifically analyzed to distinguish those reporting symptoms indicative of a TBD from those not likely to have a TBD. These analyses are presented between respondent groups and then across counties in a multi-modal comparison to official sources of data.

### 3.1. Survey Respondent Symptom Scores by Demographics

Table 1 provides demographic data for the three HMQ scoring groups: Likely to Highly Likely, Possible, and Not Likely. Out of the 260 total respondents, the majority was not likely to have a TBD. However, 22% combined were highly likely, likely, or possibly sick with a TBD. No respondents in the not likely category reported a previous diagnosis of LD.

**Table 1:** In-person survey respondent demographics, *N* = 260.

**Age**	**Percent Likely or Highly Likely** **(≥36, *n* = 18)**	**Percent Possible** **(21–35, *n* = 38)**	**Percent Not Likely** **(≤21, *n* = 204)**
18–24	0	7.9%	8.3%
25–34	5.89%	15.8%	18.6%
35–44	11.8%	10.5%	2.9%
45–54	52.9%	31.6%	26.9%
55–64	23.5%	21%	15.1%
65–74	5.9%	13.2%	2.4%
**Gender**	**Percent Likely or Highly Likely**	**Percent Possible**	**Percent Not Likely**
Male	44.4%	34.2%	52.4%
Female	55.6%	65.8%	46%
no response	0	0	1.6%
**Previous Diagnosis of Lyme Disease**	**Percent Likely or Highly Likely**	**Percent Possible**	**Percent Not Likely**
All	28%	2.6%	0%

### 3.2. Survey Respondent Symptom Scores by Demographics

Comparing the higher scoring (Likely to Highly Likely) to those in the Not Likely category, those most likely reported behaviors associated with TBD risk included tick-bite encounter recollection (61% vs. 18%) and frequent sleeping outdoors (67% vs. 29%). Twenty-eight percent of those who were deemed highly likely to have a TBD also reported a previous diagnosis of LD. These factors are under study and part of an ongoing TBD risk assessment research project for future reporting.

Figure 1 details respondents’ occupations by scoring categories. A score of 36 or above indicates a respondent is likely to have a TBD, while a score of ≥63 suggests it is highly likely. Figure 1 provides the occupations of the highest scoring respondents who are likely or highly likely to have a TBD. The top five occupational categories among the higher scoring respondents were: production occupations, homemaker, other, not specified, farm worker, and gardener. For this study, production occupations included: carpenter, scaffolding builder, construction, meat packing, petroleum field work, auto, air conditioning, or painter. For the highest scoring respondents, production occupations were limited to construction.

The most common occupations among all scoring categories were farm worker, gardener, cleaning, restaurant industry, homemaker and production occupations. The chart shows the top occupational categories of survey respondents by Possible-likely-or-Highly-likely and Not-likely categories. Respondents with higher scores indicative of LD or other TBD tended to be farmworkers or engaged in construction or other production occupations. The same was true for those with the lowest scores.

When separated by the highest scoring respondents (>36), the majority were working as farmworkers, in construction, as homemakers, or other, not specified. Of these respondents, 22% also reported working with plants and flowers, including aloe vera or citrus.

### 3.3. Symptom Severity by Respondents

The HMQ takes the symptoms most indicative of tick-borne diseases and asks respondents to report the frequency of these. They include fatigue; forgetfulness, poor short-term memory; joint pain or swelling; tingling, numbness, burning or stabbing sensations; and disturbed sleep (e.g., too much, too little, early awakening). Figure 2 presents a visual representation of the three scoring categories, with 28% of the highest scoring individuals reporting these symptoms on a daily basis. Those who possibly have, and those unlikely to have LD or other TBD, do not report having the most common debilitating symptoms on a regular basis.

Figure 3 presents mental health and health symptoms over the last 30 days by respondent scoring category. Respondents who are likely, highly likely, or possibly infected with a TBD scored higher on average than those who are unlikely to have a TBD in both overall health and mental health. Scores ranged from 1 (0–5 days poor health or mental health); 2 (6–12 days); 3 (13–20 days); and 4 (21–30 days). Those unlikely to have a TBD had lower average poor mental health and health scores than those possible to highly likely. On average, those reporting symptoms reported poor health and mental health days about five to eight days a month.

### 3.4. Triangulation of High-and-Low Scoring Respondents by Official Data Sources

All towns where survey respondents participated were contained within nine total counties. The selected towns were widely distributed throughout Texas, including South along the Mexican border, East, West, and Mid-Texas near Austin. The nine counties represented five ecosystems:

- West Gulf Coastal Plain Texas (Gregg County);
- Pecos and Stalked Plains (Dallam County);
- Edwards Plateau (Hays, Travis counties);
- South Texas Brushlands, (Hidalgo, Starr, Bexar, and Jim Wells counties);
- Oaks and Prairies Texas Ecoregion (Caldwell County).

Figure 4 offers a visual representation of counties where all survey respondents were located and counties where higher scoring survey respondents were identified. Respondents who scored over 21 are mapped by percent of total respondents in individual counties. Gregg, Travis, Hays, and Hidalgo counties had 20, 33, 37 and 20 percent of respondents reporting scores over 21, respectively, and from northeast to south. Travis, Hays, and Hidalgo were identified as higher-risk counties for the study and align with official TBD data reports. Gregg county is the outlier, with eleven percent of respondents reporting possible to highly likely TBD.

Figure 5 demonstrates percentages of those possibly, likely, or highly likely to have a TBD vs. those who are unlikely, by county. As represented in the maps above, Travis, Hidalgo, Hays, and Gregg had respondents with the most severe HMQ identified symptoms.

Given the wide variety of eco-systems in Texas, county-level comparisons provide a more structured and detailed view of higher-risk areas, which is important in public health decision making. The figures below present county-level TBD data for Texas. Additionally, as with LD, it is often unknown if cases are locally acquired or obtained out-of-state. The maps and figures below offer county-level locally acquired cases of LD, ehrlichiosis, chaffeensis, and spotted fever rickettsiosis as collected by the Zoonosis Control Branch of the Texas Department of State Health Services.

**Figure 6:**
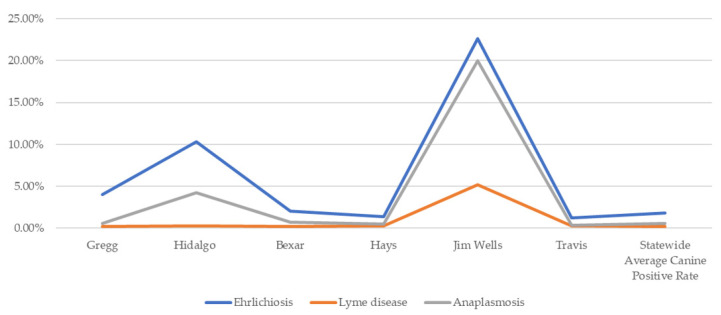
Percent of canines testing positive by TBD in selected counties (2020) [19].

In Figure 6, which documents canine cases of TBDs by county, data were not available for Dallam, Caldwell, or Star counties. Canine positive serological tests indicate disproportionately higher cases of ehrlichiosis and anaplasmosis in Hidalgo and Jim Wells counties. Gregg, Travis, Hays, Bexar, Hidalgo, and Jim Wells all had considerably higher rates of ehrlichiosis than the statewide average.

**Figure 7:**
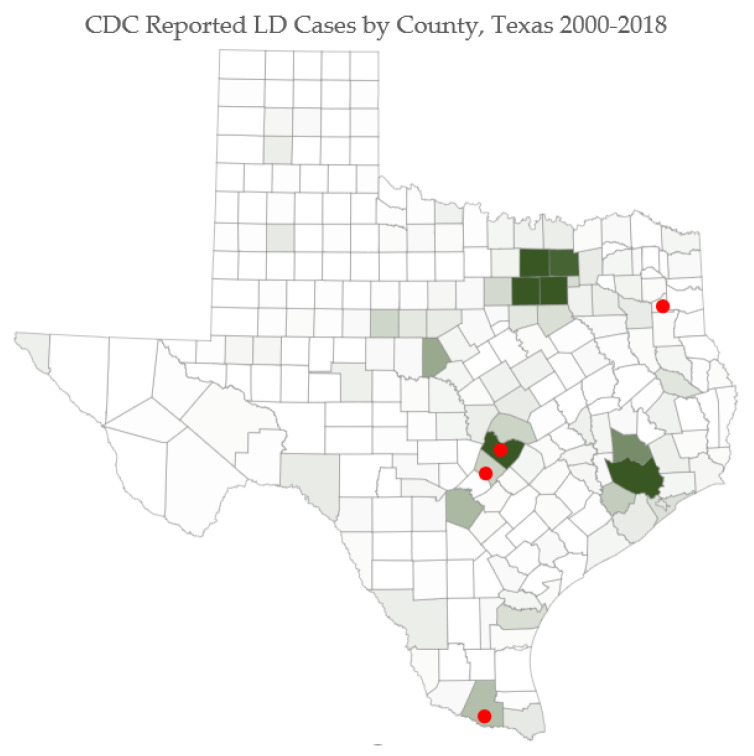
Human cases of LD, 2012–2018. County cases range from 0 to 179, with the darkest shades representing the counties with the highest number of cases. (Due to low case counts in some counties, data may not be provided to protect the identities of infected individuals).

Human LD cases are noted in Figure 7. Red dots on selected counties indicate locations where survey respondents presented with the most severe symptoms.

**Figure 8:**
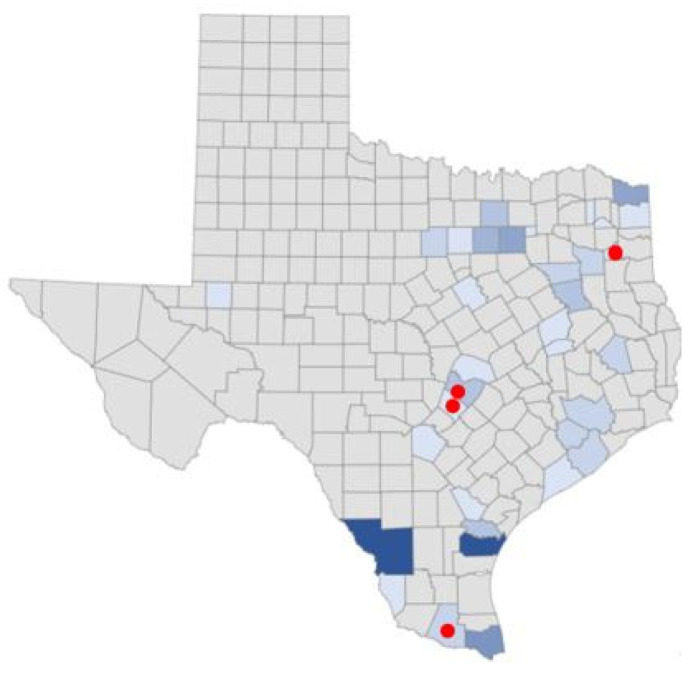
Map of DSHS locally acquired and unknown ehrlichiosis, chaffeensis, 2008–2020, by respondents with most severe symptoms.

Figure 8 is a map of human ehrlichiosis cases from data provided from the DSHS. Out-of-state-acquired cases were removed from the map. Data were not complete and did not contain all years for all counties. The map is a representation of areas where ehrlichiosis in humans is documented. Human cases fall in same ecoregions as other human TBD cases, except in the Texas panhandle ecoregion Rolling Plains, where other TBD incident reports are present. The top scoring respondent counties were represented by documented human ehrlichiosis counties.

**Figure 9:**
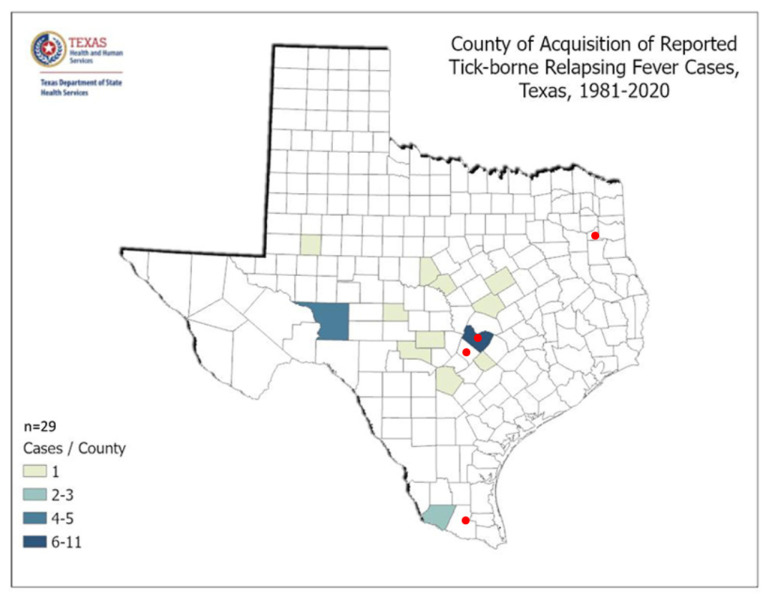
Map of DSHS locally acquired spotted fever rickettsiosis by respondents with most severe symptoms [22].

Spotted fever rickettsiosis is less common in Texas than some TBDs. However, the concentration of reported infections is found in the Oaks and Prairies ecoregion of Texas, similar to other TBDs, and where the respondents report the most severe symptoms. This ecoregion corresponds with previous studies, where rodents were found to be serologically positive for the tick-borne relapsing fever spirochete Borrelia turicatae [20]. A recent study also confirmed the spotted fever group rickettsia, Rickettsia amblyommatis, Ehrlichia chaffeensis, and Borrelia lonestari among ticks in Walker County, Texas, within the same ecoregion [21]. Figure 9 shows county acquisition of reported tick-borne Relapsing Fever cases as reported by the Texas Department of State Health Services. 

**Figure 10:**
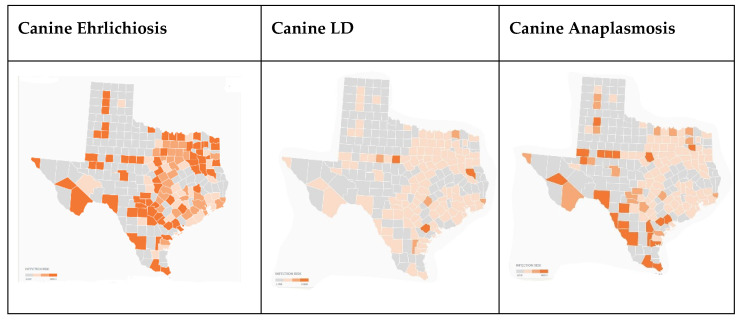
Canine ehrlichiosis, LD, and anaplasmosis by Texas counties (2021) [19].

As presented in Figure 10, canine and human reports from the CDC align in a similar manner and follow the same patterns as human cases, with fewer reports in the dryer ecosystems of west Texas, but, as with all TBDs, extending into the Texas panhandle where the ecosystem, Rolling Plains, juts out like a finger into Potter and neighboring counties. This discovery expands previous work on Texas human disease risk, but also highlights the lack of TBDs among any group in Dallam County, which is north of the Rolling Plains, and receives less rainfall and falls in ecosystem, Pecos and Staked Plains.

Ehrlichiosis cases among canines have been spreading across the Southeast and mid-west, including Texas [21,23]. In comparison to the survey respondents’ reports of higher scores in Gregg, Hays, Travis, and Hidalgo counties, the maps demonstrate similarity in case reports. Gregg, Hays, Hidalgo, and Travis counties had 4%, 1.4%, 10.3%, and 1.2% of positive ehrlichiosis canine tests. LD is less prevalent among canines across the state, with an overall state average of 0.2% positive. Anaplasmosis was also more prevalent in the survey respondent counties, but less so than ehrlichiosis. Of note, Jim Wells County had a 10.62% anaplasmosis positivity rate.

**Figure 11:**
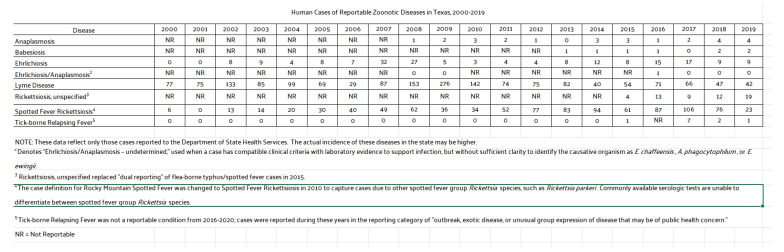
Selected human cases of TBDs in Texas from 2000 to 2019 [23].

Figure 11 presents all TBD reported cases in Texas from 2000 to 2019. There has been a noticeable uptick in ehrlichiosis cases [19,22], which positively aligns with canine serological reports. LD and spotted fever rickettsiosis are the next most prominent TBD infections in Texas. Patterns across TBDs are found primarily across central, southern, and north/east areas of the state. Exceptions are found among counties in the panhandle where one ecosystem suitable for ticks extends into the dryer region.

## 4. Discussion

Counties covered in this study included those with few or no cases of TBDs in addition to counties likely to have higher rates of TBDs as determined by canine and human reports. Findings indicate that those most likely to have a TBD were found in counties endemic to or within ecosystems supportive of tick habitats and disease risk. Overall, those reporting scores indicative of a TBD also had increased poor health and mental health days, in addition to tick-bite recall, with or without rash.

Survey respondents were located throughout Texas, covering five ecological regions. The demographics and HMQ scores revealed that a notable number of respondents were quite ill. Eight percent were likely or highly likely to have a TBD and 22% possible, likely or highly likely combined. It is not unusual for vulnerable populations to have significant health problems. In this case, eight percent reported symptoms which were severe, non-specific, and systemic and, therefore, suggestive of TBDs in a state often perceived to be non-endemic. Interestingly, almost one-third had a previous LD diagnosis. In states perceived as non-endemic, it may be unusual for medical practitioners to recognize and test for TBDs.

Survey sites were selected for their agricultural and varied ecological nature. Respondents were interviewed by Spanish-speaking nurses and community health works while on job sites, in migrant housing areas, or at community events, such as flea markets. There was no reason to suspect TBDs among the surveyed population. Using triangulation to investigate related patterns, the survey respondents were more likely to be higher scoring in counties with known higher TBD risk and official human and canine reports, noting the very small sample size in some counties, however.

Findings indicated that lower scoring unlikely survey respondents, who represent vulnerable populations in Texas, were not residing in any counties where human or canine disease risk was present. In other words, lack of illness aligned geographically with lack of TBD risk.

Respondents who were found to have a higher likelihood of a TBD were also considerably more likely to report the most common symptoms of LD and other TBDs, such as fatigue, poor short-term memory, and disturbed sleep. Those in the highly-likely scoring group also report more poor health and mental health days. Given the validity of the HMQ, those who scored as likely or highly likely to have a TBD, are clearly sick with symptoms suggestive of a TBD or other serious disease. Those who were possibly likely to have a TBD given their HMQ score generally did not have the top five symptoms “all the time” in comparison to those in higher-scoring categories. Those most likely to have a TBD were found in Hays, Hidalgo, Gregg, and Travis counties.

Triangulating canine, human TBD, and respondent symptom severity indicated overlap of zoonotic infections in key geographic areas in Texas. Medical practitioners should note the limited cases of LD and anaplasmosis among both canines and humans throughout the state, but may wish to pay close attention to human monocytotropic ehrlichiosis (HME) across the state. HME is found in South Texas [24] in areas associated with positive canine cases and where survey respondents reported higher symptom severity, as well as across the state and is now noted as a major public health threat [25].

Patterns suggest that TBD risk was highly associated with living conditions, such as sleeping outside and tick-bite encounters. Previous studies indicate that TBEs may serve as robust proxies for human disease risk [5,6]. Future studies focusing on living conditions of vulnerable populations, such as outdoor exposure, pet and livestock contact, and previous diagnosis of LD should be explored at the county level.

Triangulation and the use of a variety of data sources to uncover patterns in public health can prove useful for monitoring and evaluation applications and research. The true extent of tick-borne diseases among the U.S. population in general, and in particular among vulnerable populations, is unknown. However, public health data from the states or the CDC, in addition to canine veterinary data, allow for data triangulation to indicate patterns of potential disease risk at the county level. Triangulation can be especially useful in large states with varied ecosystems and in states perceived to be nonendemic. (In the Northeast, triangulation methods to infer patterns at the county level may serve less of a fundamental purpose, as small, endemic states have established risk patterns.)

A growing number of studies are exploring proxy data as a means for improved TBD recognition and testing, especially in areas previously considered non-endemic. Multi-modal surveillance applies triangulation of data sets across combinations of patient tick-bite encounter reports and concomitant disease, canine serological records, and entomological data identifying tick presence or established infected ticks in varying geographical locations. Additional analyses call for attention to specific ecosystems, especially in large states such as Texas, where up to ten distinct eco-systems create unique opportunities for varying vectors to thrive. As an example, Potter County Texas in the upper western panhandle lies in a small eco-region extension. Potter county lends a unique opportunity to apply multi-modal triangulation efforts, as precipitation is heavier and habitats are more suitable for tick populations than counties in adjacent eco-systems. While many of the Texas panhandle counties report zero cases of Lyme disease to the CDC, Potter is an outlier with well-established canine ehrlichiosis, anaplasmosis, and Lyme disease; patient self-reports of tick bites and associated disease; and official CDC reported cases of LD [5]. These findings point to the need for further exploration using multi-modal approaches, as no one TBD data set is fully complete. In other words, tick presence is not monitored by the CDC in every US county, and LD cases are known to be underreported by public health agencies and the CDC. 

## 5. Conclusions

An exploratory study of vulnerable individuals found distinct patterns among tick-borne diseases that algin with the highest scoring respondents. Those respondents are more likely to report feeling fatigued and very ill “all of the time.” Given the ongoing national debates regarding LD among those who are clinically diagnosed, this study in addition to previous research on TBDs in Texas, suggests that public health may be wise to note the highest likelihood of disease in their respective counties, paying close attention to the wide variety of ecosystems and nonspecific symptoms associated with a variety of zoonotic infection.

## 6. Limitations

Patient reports of tick bites and diagnoses present challenges, as patients may be mis-diagnosed or lack proper memory recall. This study was not designed to capture statistically significant findings, as county-level data on most TBDs are generally not fully available. Although the Texas DSHS responded to requests for information, data were often not reportable or not available for all years. Official data from state health services rely on passive surveillance and reporting from medical practitioners. Not all cases are reported due to a lack of testing or reporting. Triangulating patterns, therefore, was the focus of the study, but the methodology itself limits definitive conclusions. Many respondents report country of origin as Mexico, with full-time residence in Texas. Case reports demonstrate a diverse group of Borrelia species found among LD patients from Mexico [26], as well as Bison [27], and a notable amount of rickettsia in canines in Northeastern Mexico [28].

The sample size of respondents likely to have a TBD was also small. Future studies should include more detailed personal information regarding respondents, their health, and living conditions that might indicate likely exposure to ticks. Other states with varied ecosystems and expanding TBDs among humans and canines should also be explored to assess similar patterns at the county level. These states should also include counties with vulnerable populations who may not have reliable access to medical care. Laboratory confirmation also would be useful, along with survey responses.

## Data Availability

Data available upon request.
